# A SCARECROW-based regulatory circuit controls *Arabidopsis thaliana* meristem size from the root endodermis

**DOI:** 10.1007/s00425-016-2471-0

**Published:** 2016-02-05

**Authors:** Laila Moubayidin, Elena Salvi, Leonardo Giustini, Inez Terpstra, Renze Heidstra, Paolo Costantino, Sabrina Sabatini

**Affiliations:** Laboratory of Functional Genomics and Proteomics of Model Systems, Dipartimento di Biologia e Biotecnologie, Università La Sapienza, P.le Aldo Moro, 5, 00185 Rome, Italy; Crop Genetics Department, John Innes Centre, Norwich Research Park, Norwich, NR4 7UH UK; Section Molecular Genetics, Department of Biology, Faculty of Science, Utrecht University, Padualaan 8, 3584 CH Utrecht, The Netherlands; Faculty of Science, SILS, University of Amsterdam, POSTBUS 94215, 1090 GE Amsterdam, The Netherlands; Plant Developmental Biology, Wageningen University and Research Centre, Droevendaalsesteeg 1, 6708 PB Wageningen, The Netherlands

**Keywords:** ARABIDOPSIS RESPONSE REGULATOR 1 (ARR1), Differentiation, Gibberellin, Hormones, Root, SCARECROW (SCR)

## Abstract

**SCARECROW controls Arabidopsis root meristem size from the root endodermis tissue by regulating the DELLA protein RGA that in turn mediates the regulation of*ARR1*levels at the transition zone.**

Coherent organ growth requires a fine balance between cell division and cell differentiation. Intriguingly, plants continuously develop organs post-embryonically thanks to the activity of meristems that allow growth and environmental plasticity. In *Arabidopsis thaliana*, continued root growth is assured when division of the distal stem cell and their daughters is balanced with cell differentiation at the meristematic transition zone (TZ). We have previously shown that at the TZ, the cytokinin-dependent transcription factor ARR1 controls the rate of differentiation commitment of meristematic cells and that its activities are coordinated with those of the distal stem cells by the gene *SCARECROW* (*SCR*). In the stem cell organizer (the quiescent center, QC), SCR directly suppresses *ARR1* both sustaining stem cell activities and titrating non-autonomously the *ARR1* transcript levels at the TZ via auxin. Here, we show that SCR also exerts a fine control on *ARR1* levels at the TZ from the endodermis by sustaining gibberellin signals. From the endodermis, SCR controls the RGA REPRESSOR OF ga1-3 (RGA) DELLA protein stability throughout the root meristem, thus controlling *ARR1* transcriptional activation at the TZ. This guarantees robustness and fineness to the control of ARR1 levels necessary to balance cell division to cell differentiation in sustaining coherent root growth. Therefore, this work advances the state of the art in the field of root meristem development by integrating the activity of three hormones, auxin, gibberellin, and cytokinin, under the control of different tissue-specific activities of a single root key regulator, SCR.

**Electronic supplementary material:**

The online version of this article (doi:10.1007/s00425-016-2471-0) contains supplementary material, which is available to authorized users.

## Introduction

The ability of plants to generate organs continuously depends on the activity of meristems, localized regions where plant stem cells reside (reviewed in Heidstra and Sabatini 2014). The root meristem is responsible for generating the root system, which anchors the plant to the soil while providing all the necessary nutrients. Longitudinally, the root meristem can be divided into three functionally distinct zones: a distal stem cell niche (SCN) generating transit-amplifying cells, which divide in the proximal meristem (PM) and differentiate in the elongation/differentiation zone (EDZ) (reviewed in Heidstra and Sabatini 2014). The boundary where cells leave the meristem and enter the EDZ is called transition zone (TZ). To maintain root meristem activity and thus root growth, the activities of these zones must be well coordinated, and different plant hormones have been shown to play a crucial role in ensuring this coordination. For example, the balance between cell division in the PM and cell differentiation in the EDZ depends on the antagonistic interaction between cytokinin, promoting cell differentiation, and auxin, prompting cell division (Dello Ioio et al. 2007, 2008). Cytokinin promotes cell differentiation via the cytokinin-dependent response transcription factor *ARABIDOPSIS RESPONSE REGULATOR 1* (*ARR1*) (Dello Ioio et al. 2007; Taniguchi et al. 2007), which in turn activates the auxin signaling inhibitor gene *SHY2* (Tian et al. 2002; Dello Ioio et al. 2008). Another plant hormone, gibberellin (GA) (Olszewski et al. 2002; Hedden and Sponsel 2015), has been reported to control cell division in the PM acting from the root endodermis (Achard et al. 2009; Ubeda-Tomás et al. 2009). In particular, it was shown that GA sustains cell division by suppressing the *ARR1* transcript level at the TZ via the DELLA protein REPRESSOR OF GA1-3 (RGA) (Silverstone et al. 1998; Moubayidin et al. 2010).

A single gene, *SCARECROW* (*SCR*) (Di Laurenzio et al. 1996), is responsible for the spatial coordination between stem cell activity in the SCN and differentiation of their daughters at the TZ (Moubayidin et al. 2013). The SCR protein, a member of the GRAS family of transcription factors, is specifically expressed in the stem cell niche organizing cells, the quiescent center (QC), and in the endodermis (Di Laurenzio et al. 1996; Pysh et al. 1999; Wysocka-Diller at al. 2000; Lee et al. 2008). Besides its role in sustaining the stem cells’ self-renewal activity (Sabatini et al. 2003; Moubayidin et al. 2013) SCR is known to be involved in many other root developmental events such as regulation of formative stem cell division (Heidstra et al. 2004; Cui et al. 2007; Sozzani et al. 2010; Cruz-Ramírez et al. 2012) and protoxylem formation (Carlsbecker et al. 2010).

SCR sustains stem cell activities by directly suppressing *ARR1* expression in the QC cells, thus titrating auxin production (Moubayidin et al. 2013). Auxin produced in the QC not only controls stem cell division activity but at the same time acts, via polar transport, as a long-distance signal to fine-tune the level of *ARR1* transcription in the TZ (Moubayidin et al. 2013). In this way, ARR1 is positioned at the TZ where cell differentiation is initiated (Moubayidin et al. 2013).

Here, we report that in addition to controlling ARR1 position and activity via auxin (Moubayidin et al. 2013) SCR controls *ARR1* levels also through GA from the endodermis, thus contributing to the fine-tuning of the ARR1-mediated cell differentiation commitment which is necessary to control root meristem size.

## Materials and methods

### Plant materials, growth conditions, and treatments

The *Arabidopsis thaliana* ecotypes Columbia (Col-0), Wassilewskija (Ws) and Landsberg *erecta* (Ler) were used. *arr1*-*3* is in Col-0 background (Dello Ioio et al. 2007), *scr*-*1* and *sne*-*1* are in Ws background (Cui and Benfey 2009; Moubayidin et al. 2013), *rga*-*24* is in Ler background (Moubayidin et al. 2010), *pRGA*:*rga*-*Delta17* and *pRGA*:*GFP*:*rga*-*Delta17* are in Ler background (Dill et al. 2001), *ARR1*:*GUS*, *scr*-*1 ARR1*:*GUS* and *RGA*:*GFP* were previously described (Dill et al. 2001; Mason et al. 2004; Moubayidin et al. 2010, 2013). Seeds were sterilized and grown as described previously (Perilli and Sabatini 2010). Gibberellin (GA_3_-Sigma) treatments were performed using 10 μM GA dissolved in ethanol, for 24 h on 5-day-old seedlings as previously described (Moubayidin et al. 2010). Experiments with *scr*-*3 N9094 UAS*::*SCR*:*GR* and relative control plants were performed by germinating seeds on plates containing 2 μM Dexamethasone (Dex-Sigma) for 5 days and then transferring the seedlings on plates containing 10 μM GA and 2 μM Dex for 24 h. *pRGA*:*rga*-*Delta17* and *pRGA*:*GFP*:*rga*-*Delta17* were obtained from Prof Tai-Ping Sun, Department of Biology, Duke University, Durham, NC, USA. *sne*-*1* mutant (FLAG_461E03) was obtained from the Versailles Arabidopsis Stock Center, INRA, France.

### Root length and meristem size analysis

For root length measurements, plates were photographed and the resulting images were analyzed using the ImageJ software available online (http://rsbweb.nih.gov/ij/) as described in Perilli and Sabatini (2010). Root meristem size is expressed as the number of cortex cells in a file extending from the quiescent center to the first elongated cortex cell, as described previously (Dello Ioio et al. 2007; Perilli and Sabatini 2010).

At the optical microscope, the distinction between dividing and differentiating root meristematic cells belonging to the cortex file is performed by the operator taking into account multiple parameters, i.e., the smaller size, the presence of a large central vacuole, and the denser cytoplasm of dividing meristematic cells versus differentiating ones. Moreover, both the position of the TZ in the outward tissue, the epidermis, and the extension of the lateral root cap are also evaluated as helpful references to univocally identify the cortex TZ and, hence, to measure meristem size. For each experiment, a minimum of 90 plants was analyzed using an Axio Imager.A2 (Zeiss) light microscopy and average and standard deviation were calculated. To measure root development over time, root length and meristem size were analyzed at different days post-germination (dpg): 3 dpg, 5 dpg, 7 dpg, 9 dpg, 12 dpg, and 15 dpg.

### GUS histochemical assay

To visualize *ARR1*:*GUS* line in wild-type and mutant backgrounds, GUS histochemical assay was performed using the β-glucuronidase substrate X-gluc (5-bromo-4-chloro-3-indolyl glucuronide, Duchefa) as previously described (Perilli and Sabatini 2010; Moubayidin et al. 2013). Five-day-old seedlings were incubated for 16 h at 37 °C in the dark and imaged using the Axio Imager.A2 (Zeiss) microscopy.

### qRT-PCR experiments

Total RNA was extracted from 5 days-old roots using the TRIsure reagent (Bioline), and the first strand cDNA was synthesized using the Superscript^®^ III First Strand Synthesis System (Invitrogen). PCR amplification was carried out in the presence of the double-strand DNA-specific dye SYBR Green (Sigma). Amplification was monitored in real time with the 7300 Real-Time PCR System (Applied Biosystems). Experiments were performed in triplicates from RNA of root tissue. Amplification of *ACTIN 2* served as control. Data are expressed in Log2 (2^−ΔΔCt^) ratio. qRT-PCR was performed three times on three independent RNA batches, and results were comparable in all experiments. Student *t* test was performed to know the actual significance of these data, the site used is: http://graphpad.com/quickcalcs/ttest2.cfm. Quantitative RT-PCR analysis was conducted using the following gene-specific primers:

ARR1 FW: 5′-TTGAAGAAACCGCGTGTCGTCT-3′ and ARR1 RV: 5′-CCTTCTCAACGCCGAGCTGATTAA-3′ for *ARR1* (Moubayidin et al. 2013)

SNE FW: 5′-GTTCACCATGTCGTCGGAGA-3′ and

SNE RV: 5′-GAGCTCTGTTTCCGACAAGTG-3′ for *SNEEZY* (Sozzani et al. 2010)

RGA FW: 5′-CATGTTCCTCCACCGTCTTC-3′ and

RGA RV: 5′-AAAAAGGCAAAACCCTAGATC-3′ for *RGA*

ACT FW: 5′-CCTTCTCAACGCCGAGCTGATTAA-3′ and

ACT RV: 5′-GTGGATTCCAGCAGCTTCCAT-3′ for *ACTIN2*

### Molecular cloning and genotyping

The SCR coding sequence lacking the stop codon was combined with the glucocorticoid receptor (GR) and cloned in the upstream activating sequence (UAS) cassette in between the *6xUAS* and 35STerminator. Using the flanking NotI sites the *UAS*::*SCR* cassette was subsequently cloned into the pGreenII (Hellens et al. 2000)-based pGII124 binary vector (methotrexate resistance in planta) and transformed into *scr*-*3* N9094 plants by floral dip.

The primers for genotyping plants for *rga*-*24* deletion line were as follows:

RGA n212: 5′-GGTGATTTTCACGGTGGTTG-3′

*rga*-*24* n205: 5′-TCGCTTAGTAGTTAGTACTC-3′

*rga*-*24* n253: 5′-CATAGACCATAGTATTCGTGA-3′

RGA n212 and *rga*-*24* n205 pair was used for amplifying the wild-type DNA copy of RGA, while *rga*-*24* n205 and *rga*-*24* n253 were used to amplify DNA from the mutant as described in de Lucas et al. (2008).

### Confocal image processing

Confocal images of median longitudinal sections of 5-day-old roots were taken using a Zeiss LSM 780 microscope. A 10 mg/ml propidium iodide (Sigma) solution was used to visualize the cell wall.

## Results

### SCR mediates GA-dependent control of root meristem size from the endodermis

**Fig. 1:**
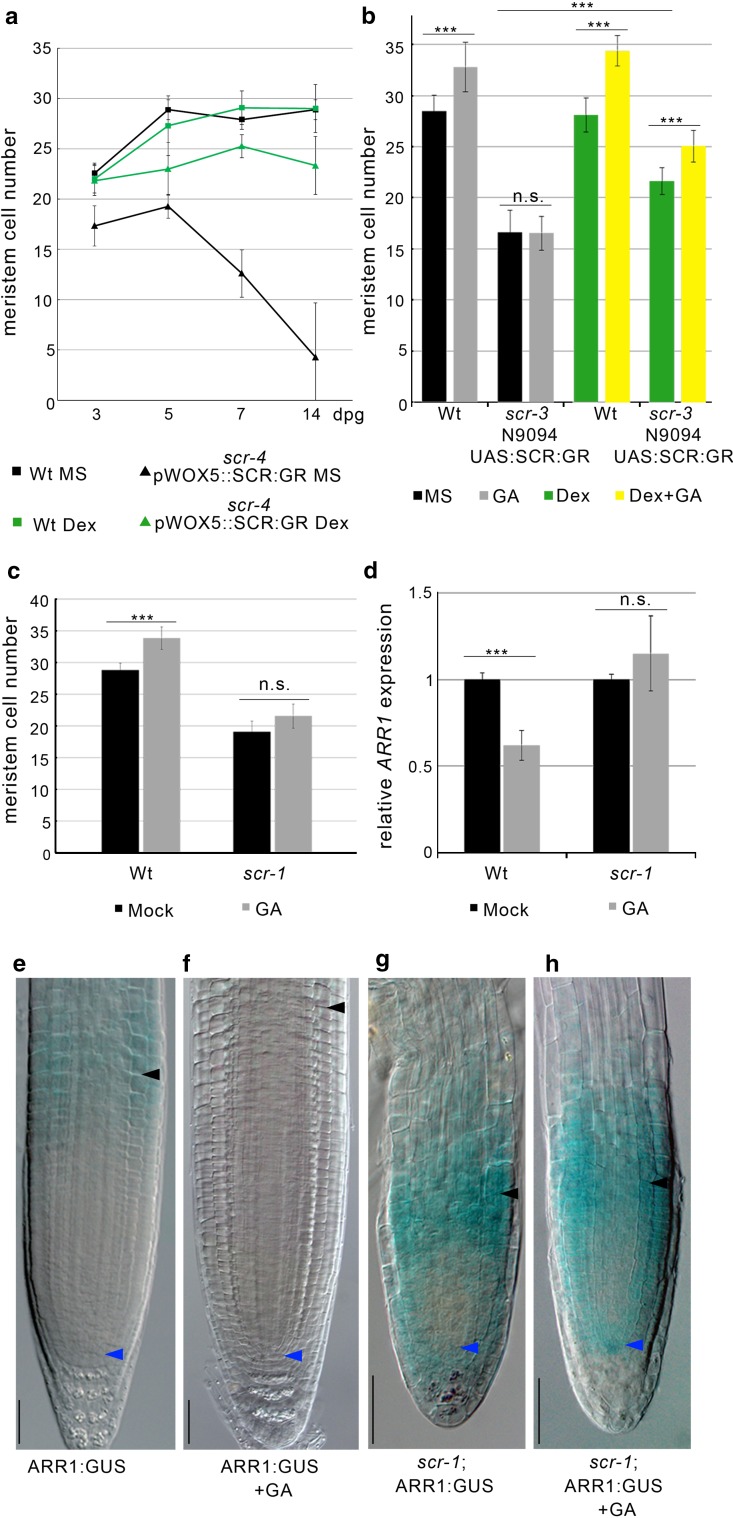
SCR controls *ARR1* expression via gibberellin from the endodermis. **a** Root meristem cell number of wild type (Wt) and *scr*-*4 pWOX5*:*UGU*-*GFP UAS*:*SCR*:*GR* (*scr*-*4* pWOX5::SCR:GR) treated (*green lines*) or untreated (*black lines*) with Dexamethasone (Dex) measured over time. *dpg* days post-germination. *Error bars* indicate standard deviation. **b** Root meristem cell number of Wt and *scr*-*3* N9094 *UAS*::*SCR*::*GR* grew on MS (*black columns*) for 5 days treated for 24 h with 10 µM Gibberellin (GA, *gray columns*), 2 µM Dex (*green columns*), and both (Dex + GA, *yellow columns*). *Error bars* indicate standard deviation. Student’s *t* test; ***P < 0.001; *n.s.* not significant. **c**, **d** Root meristem cell number (**c**) and qRT-PCR of *ARR1* expression (**d**) in 5 dpg wild-type and *scr*-*1* roots grown 24 h on 10 µM gibberellin (GA) (*gray columns*) or mock (*black columns*). *Error bars* indicate standard deviation. Student’s *t* test; ***P < 0.001; n.s., not significant (*n* = 3 for qRT-PCR experiments). **e**–**h** Expression of the *pARR1*:*ARR1*:*GUS* construct in root meristems at 5 dpg of wild type (**e**, **f**) and of *scr*-*1* (**g**, **h**) grown on mock (**e**, **g**) and 24 h on 10 µM GA (**f**, **h**). *Blue and black arrowheads* indicate the quiescent center and the cortex transition zone, respectively. *Scale bars* represent 50 µm

Tissue-specific complementation experiments where the SCR protein was reintroduced in the *scr* mutant only in specific districts of its expression domain suggest that activity of SCR in the QC is necessary and sufficient to control QC and stem cell function: in *scr* mutant plants where SCR was expressed only in the QC, stem cell activities and root growth were reestablished (Sabatini et al. 2003; Moubayidin et al. 2013). However, roots were shorter and root meristems smaller than wild type (Sabatini et al. 2003, Fig. 1a). Ground tissue (cortex and endodermis)-specific complementation could not sustain root meristem indeterminate growth but roots grew longer (Sabatini et al. 2003) and meristems were bigger compared to the *scr* mutant roots (Fig. 1b), indicating that SCR expression in the endodermis, besides regulating root radial patterning (Di Laurenzio et al. 1996; Heidstra et al. 2004) contributes also to root meristem size determination and overall root growth.

GA is predicted to regulate root meristem size from the endodermis (Achard et al. 2009; Ubeda-Tomás et al. 2009) and in this tissue, SCR is known to directly activate the *SNEEZY* (*SNE*) gene (Cui et al. 2007), one of the F-box proteins necessary for gibberellin-mediated degradation of the RGA protein (Ariizumi and Steber 2011; Ariizumi et al. 2011). We thus wondered whether SCR controls root meristem size from the endodermis sustaining GA activity. To this aim we first exposed wild-type and *scr*-*1* mutant seedlings to exogenous GA application, monitoring root meristem size after 24 h.

Root meristem size was measured as the number of meristematic cortex cells using manual labeling at the optical microscope (see “Materials and methods”) rather than the program Cell-o-Tape (French et al. 2012). In fact, while automating and speeding up operations, this program does not take into account changes neither in the density of the cytoplasm nor in the vacuole size of differentiating cells, important characteristics that allow, in our experience, a more precise positioning of the transition zone (Perilli and Sabatini 2010).

Upon GA treatments wild-type root meristem were enlarged, while no increase in meristem size was observed in the *scr*-*1* mutant (Fig. 1c, e–h) suggesting that SCR activity is necessary to mediate GA-dependent control on root meristem size. To understand whether SCR functions in the endodermis to control root meristem size via GA we expressed the *SCR* gene in this tissue of the *scr*-*3* mutant roots using a GAL4/UAS trans-activation system. In wild-type roots the N9094 (*GAL4,UAS∷GFP)* enhancer trap line from the J. Haseloff (http://www.plantsci.cam.ac.uk/Haseloff) collection, *GFP*, is expressed specifically in the cortex and endodermis tissues (Suppl. Fig. S1a) while in the *scr* mutant it is expressed in the monolayer typical of this mutant (Suppl. Fig. S1b) (Sabatini et al. 2003). The N9094 driver was introduced in *scr*-*3* mutant plants carrying a *UAS*::*SCR*::*GR* construct where the SCR protein activities are inducible by Dexamethasone (Dex). In the resulting *scr*-*3* N9094 *UAS*::*SCR*::*GR*(*N9094*≫*SCR*:*GR*) plants, *GFP* expression monitored GAL4 activity and hence ectopic *SCR*::*GR* expression in the mutant background. *scr*-*3N9094*≫*SCR*:*GR* plants were germinated on Dex and were transferred on GA for 24 h. Analysis of the root meristem size of these plants revealed that the endodermis-specific induction of SCR was sufficient to enlarge the *scr*-*3* meristem (Fig. 1b). Moreover, GA sensitivity was restored, as the meristem enlarged compared to GA-treated *scr*-*3* roots (Fig. 1b). These data substantiate the notion that SCR mediates GA-dependent control of root meristem size functioning from the endodermis.

### SCR mediates GA-dependent *ARR1* transcriptional suppression

We have previously shown that GA controls root meristem size by lowering the expression of *ARR1* at the TZ (Moubayidin et al. 2010) (Fig. 1c–f). We thus wondered whether SCR promotes the GA-mediated downregulation of *ARR1* expression at the TZ, in addition to the regulation it has been shown to exert from the QC via auxin (Moubayidin et al. 2013).

Indeed, while GA negatively controls *ARR1* transcription in the wild type (Fig. 1d–f) (Moubayidin et al. 2010), no downregulation of *ARR1* expression was observed in the *scr*-*1* mutant upon GA treatment as visualized both by quantitative real-time PCR (qRT-PCR) (Fig. 1d) and the analysis of *scr*-*1* plants harboring a *pARR1*:*ARR1*:*GUS* (Fig. 1g, h). These data suggest that SCR is necessary to mediate the GA-dependent transcriptional downregulation of *ARR1* in the control of root meristem size.

### SCR mediates *ARR1* transcriptional suppression via the RGA protein

Since GA acts via RGA to suppress *ARR1* (Moubayidin et al. 2010) and SCR has been shown to directly control transcription of the specific RGA F-box protein SNE (Cui et al. 2007; Ariizumi and Steber 2011; Ariizumi et al. 2011), we hypothesized that the inability of GA to down-regulate *ARR1* in the *scr*-*1* mutant background could be due to increased levels of the RGA protein in the mutant.

**Fig. 2:**
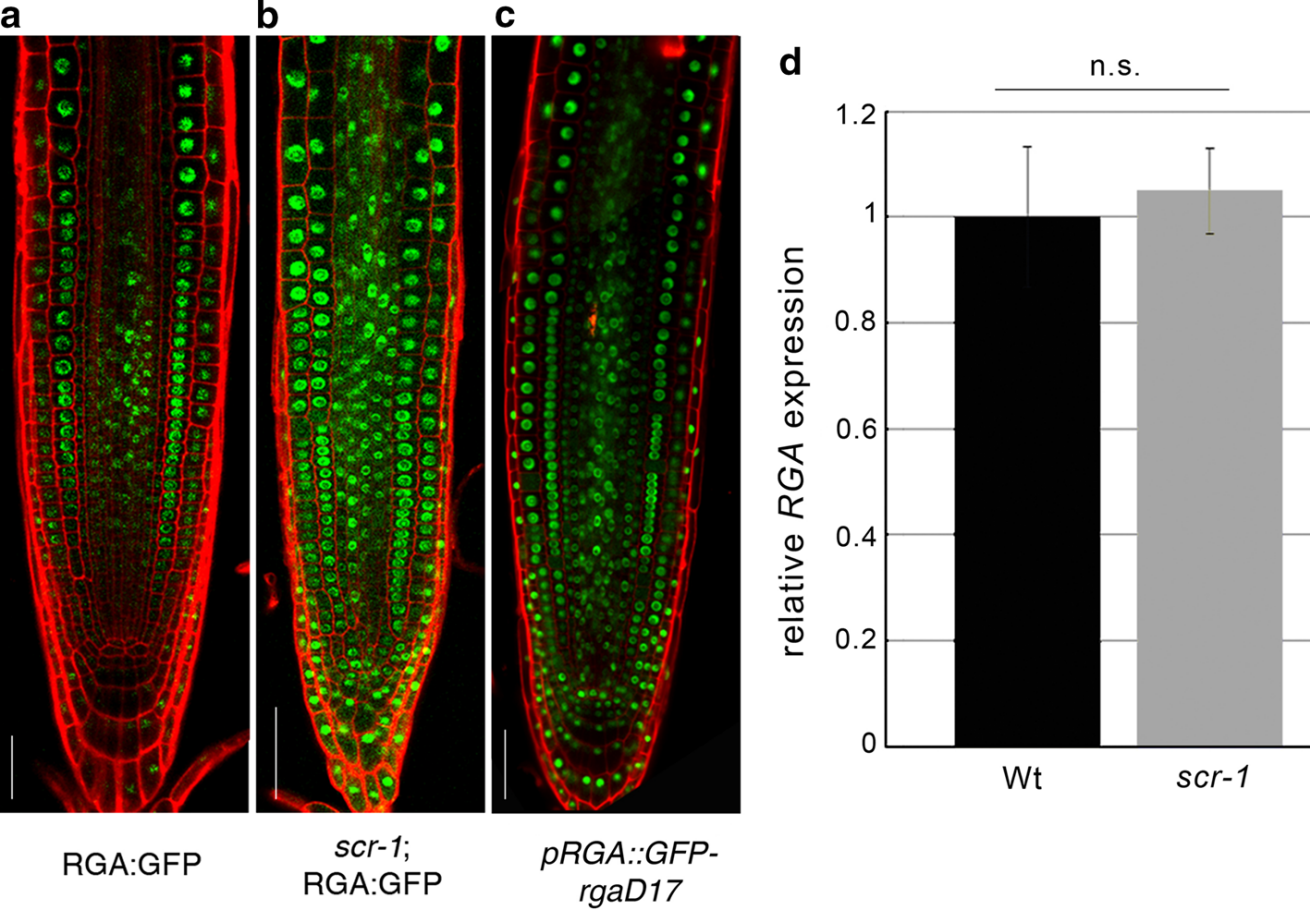
*SCR* controls *RGA* protein stability in the root meristem. **a**, **b** Expression of the *pRGA*:*RGA*:*GFP* (RGA:GFP) construct in wild-type roots (Wt) (**a**) and in the *scr*-*1* mutant (**b**). **c** Root meristem expression of pRGA:GFP:rga-Delta17 (pRGA::GFP-rgaD17). *Scale bars* represent 50 µm. **d** qRT-PCR of *RGA* expression in 5 dpg wild-type and *scr*-*1* roots. *Error bars* indicate standard deviation. Student’s *t* test; n.s., not significant, *n* = 3

**Fig. 3:**
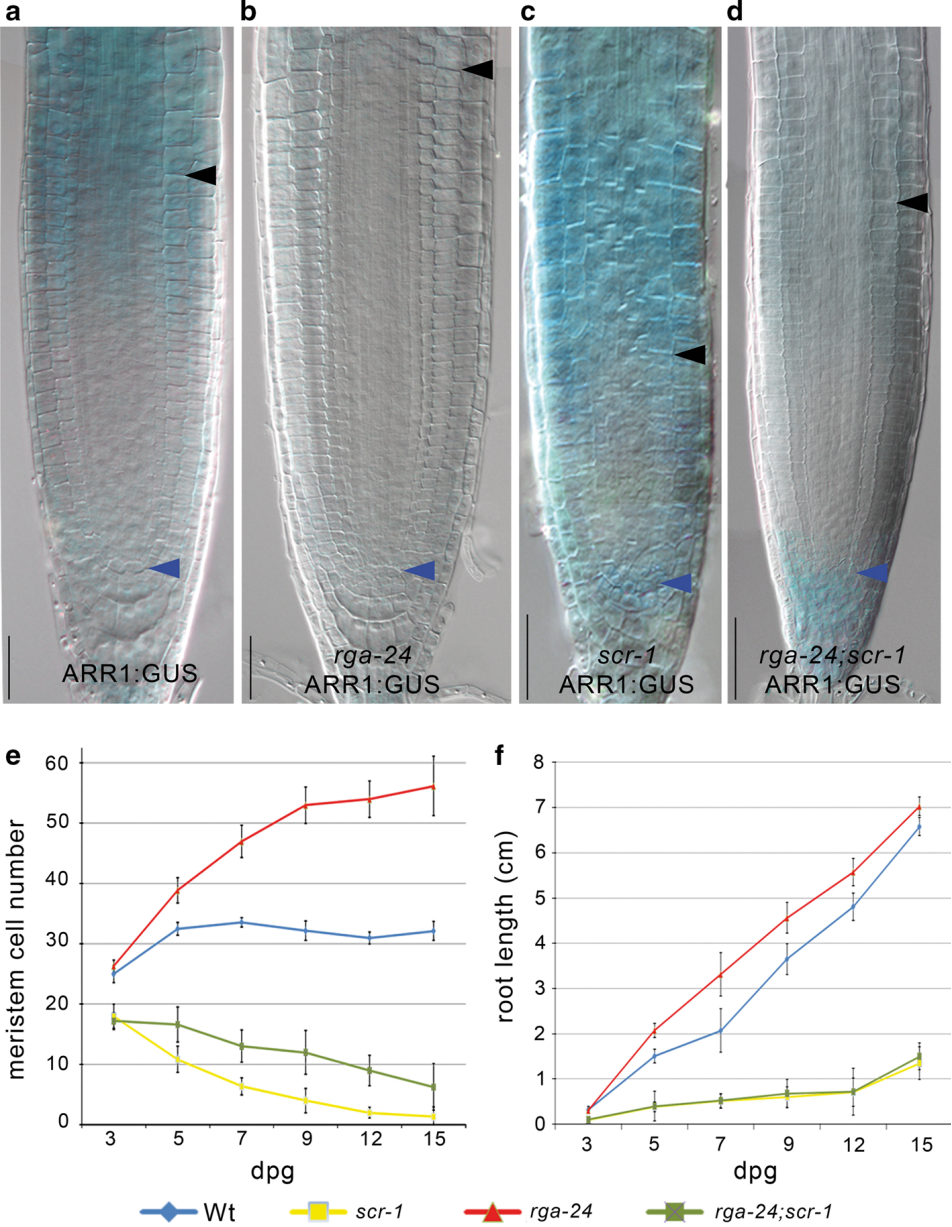
SCR controls *ARR1* expression at the transition zone via the DELLA protein RGA. **a**–**d** Expression of the *pARR1*:*ARR1*:*GUS* construct in Wt (**a**), *rga*-*24* (**b**), *scr*-*1* (**c**), and *rga*-*24;scr*-*1* (**d**) mutants. Roots were analyzed 5 days post-germination (dpg). *Scale bars* represent 50 µm. **e**, **f** Root meristem cell number (**e**) and root length (**f**) measured over time of Wt, *scr*-*1,* *rga*-*24*, and *rga*-*24;scr*-*1*

To test this hypothesis, we monitored RGA expression in the *scr*-*1* mutant using a *pRGA*:*RGA*:*GFP* translational fusion (Silverstone et al. 2001). As predicted, the level of the RGA protein was higher than in wild-type roots, and the domain of RGA expression was extended to the whole meristem in *scr*-*1*, including the stem cell niche (Fig. 2a, b). In contrast, the level of the *RGA* transcript was not increased in the *scr*-*1* mutant as revealed by qRT-PCR (Fig. 2d), suggesting that SCR controls RGA at the protein level. Accordingly, the translational fusion of the GA-insensitive semi-dominant mutant version of RGA protein under its own promoter and fused to the GFP, named *rga*-*Delta17(pRGA*:*GFP*:*rga*-*Delta17)* (Dill et al. 2001), was found to be expressed throughout the meristem (Fig. 2c), thus phenocopying the ectopic expression of RGA shown in *scr*-*1* mutant (Fig. 2b). We next set to demonstrate how, in the *scr*-*1* mutant, the high level of RGA (Fig. 2b) contributes to the high level of *ARR1* expression (Figs. 1g and 3c) (Moubayidin et al. 2013). To this end, we generated *rga*-*24*;*scr*-*1* double-mutant plants—where *rga*-*24* is the loss of function mutant of RGA (Dill and Sun 2001)—carrying a *pARR1*:*ARR1*:*GUS* construct. As previously shown ARR1 levels in the *rga*-*24* mutant background were low compared to the wild-type roots (Moubayidin et al. 2010) (Fig. 3a, b), while ARR1 was ectopically expressed in the QC of the *scr*-*1* mutant (Fig. 3c) (Moubayidin et al. 2013). Compared to *scr*-*1*, ARR1 expression in *rga*-*24*;*scr*-*1* plants was lower in the TZ but unaltered in the QC (Fig. 3c, d). This pattern of expression of ARR1 is consistent with the notion that, in the QC, SCR directly controls *ARR1* (Moubayidin et al. 2013), while at the TZ, SCR modulates *ARR1* expression cell non-autonomously via RGA. Interestingly, *rga*-*24*;*scr*-*1* plants displayed a slightly longer root meristem than *scr*-*1* plants but could not sustain indeterminate root growth (Fig. 2e, f), resembling *scr* mutant plants where SCR function was reintroduced only in the endodermis (Sabatini et al. 2003). Indeed, the stem cell niche was not rescued as visualized by the presence of differentiation markers in place of columella stem cells (Suppl. Figs S1c-S1f).

### Post-translational regulation of RGA controls root meristem size

**Fig. 4:**
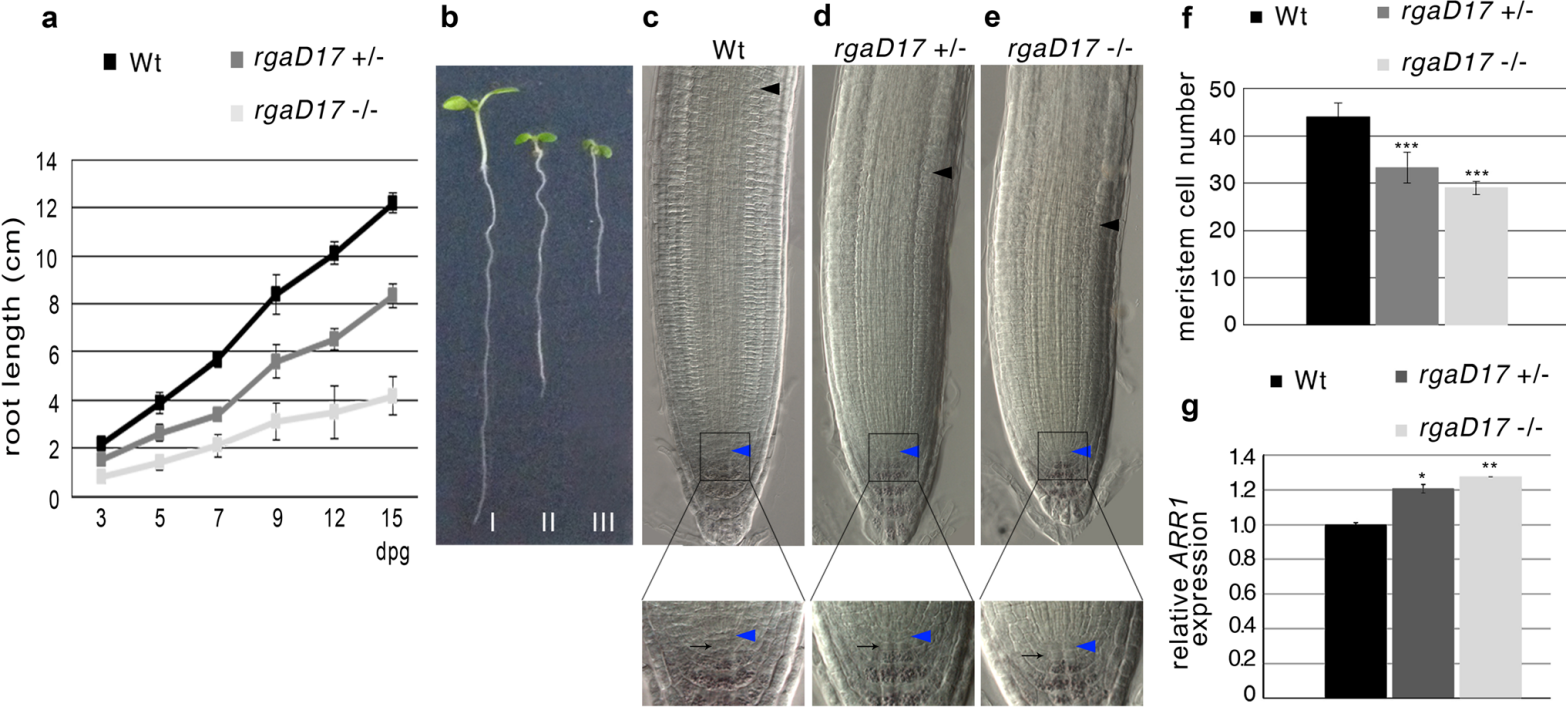
Post-translational regulation of RGA controls root meristem size. **a** Root length measured over time of Wild type (Wt), *pRGA*::*rga*-*Delta17* (*rgaD17*) heterozygote (±) and homozygote (−/−) mutants. **b** Seedlings of Wt (I), *rgaD17* ± (II), and *rgaD17* −/− (III) mutants. **c**–**e** Root meristem of 5-day-old Wt, *rgaD17* heterozygote (±) and homozygote (−/−) mutants. *Blue and black arrowheads* indicate the QC and the cortex TZ, respectively. Blow up of the stem cell niches and lugol staining is shown in the *bottom* panel. *Black arrows* indicate the columella stem cells. **f**, **g** Root meristem cell number (**f**) and qRT-PCR of *ARR1* expression (**g**) in 5 dpg Wt, *rgaD17* heterozygote (±) and homozygote (−/−) mutants roots. *Error bars* indicate standard deviation. Student’s *t* test; ***P < 0.001; **P < 0.01; *P < 0.05 (*n* = 3 for qRT-PCR experiments)

**Fig. 5:**
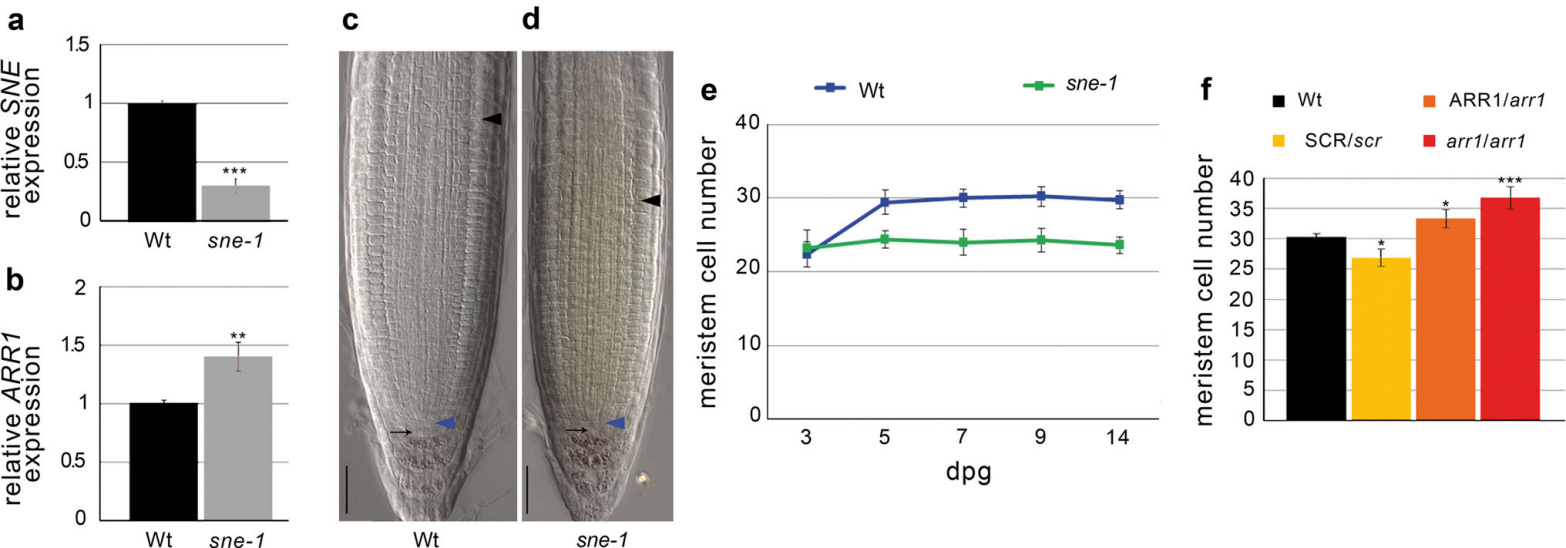
Modulation of *ARR1* level affects root meristem size. **a**, **b** qRT-PCR of *SNE* (**a**) and *ARR1* (**b**) expression in 5dpg roots in wild-type and *sne*-*1* background. *Error bars* indicate standard deviation. Student’s *t* test; **P < 0.01; ***P < 0.001, *n* = 3. **c**, **d** Root meristems of wild type (**c**) and *sne*-*1* mutant (**d**) at 5 dpg. *Blue and black arrowheads* indicate the QC and the cortex TZ, respectively. **e** Root meristem cell number of wild type and *sne*-*1* measured over time. *Error bars* indicate standard deviation. **f** Root meristem cell number measured at 5dpg of Wt roots, heterozygote SRC/*scr*-*1* and ARR1/*arr1*-*3* roots and homozygote *arr1*-*3* roots. *Error bars* indicate standard deviation. Student’s *t* test; *P < 0.05; ***P < 0.001

It has been shown that SCR directly activates *SNE* (Cui et al. 2007), an F-box responsible for DELLA protein degradation (Ariizumi and Steber 2011; Ariizumi et al. 2011). Moreover, we found that the degradation-resistant protein version of the RGA protein leads to an ectopic expression of RGA as shown in *scr*-*1* (Fig.s 2a-2c). Therefore, we wondered whether SCR could regulate the RGA-dependent control of root meristem size by acting on RGA protein stability. First, we analyzed the rate of root growth and the meristem size of *pRGA*::*rga*-*Delta17* (*rga*-*Delta17*) (Dill et al. 2001). *rga*-*Delta17* heterozygote and homozygote seedlings, showed a dose-dependent phenotype compared to the control plants, as the roots grew shorter with increasing doses of *rga*-*Delta17* copies (Fig. 4a, b). Accordingly, the root meristem of 5-day-old heterozygote and homozygote *rga*-*Delta17* mutants showed a reduction in their size in a dose-dependent manner respect to the control, without affecting SCN activity (Fig. 4c–f). To link this gradual reduction in root length and meristem size to an enhancement in cell differentiation rate, we analyzed mRNA *ARR1* levels in heterozygote and homozygote *rga*-*Delta17* roots. We found that the content of *ARR1* was inversely proportional to the root lengths, as ARR1 increased with increasing copies of the mutated version of RGA (Fig. 4g). All together these data showed that the GA-mediated degradation control of RGA is sufficient to fine-regulate root meristem size and root growth. Consequently, we predicted a similar effect on root meristem size and root growth control in plant mutated in the *SNE* gene. To this end, we used the *sne*-*1* loss of function mutant (Cui and Benfey 2009) where the transcriptional level of *SNE* is severely reduced (Fig. 5a), and analyzed the *ARR1* mRNA level by qRT-PCR. As visualized in Fig. 5b, the *ARR1* transcript level was higher in the *sne*-*1* mutant background than in the wild type, supporting the idea that the SNE protein is necessary for an indirect control of *ARR1* expression. We then wondered whether the high level of *ARR1* correlates with an increased differentiation rate at the TZ resulting in a shorter root meristem of the *sne*-*1* mutant. Indeed the root meristem of the *sne*-*1* mutant was smaller compared to wild type (Fig. 5c d). Moreover, *sne*-*1* root meristem size is precociously imposed (Fig. 5e) according to the role of GA in controlling *ARR1* transcript levels (Moubayidin et al. 2010), suggesting that the SNE protein is involved in root meristem size determination, presumably by controlling *ARR1* activity.

### ARR1 controls root meristem size in a dose-dependent manner

The data presented above suggest that the levels of *ARR1* must be very finely regulated to determine root meristem size. We, therefore, set to establish whether a dose-dependent relation exists between the level of *ARR1* and root meristem size by measuring the meristems of plant heterozygous for the *scr*-*1* and *arr1* mutations obtained by backcrossing the *scr* and *arr1* mutants with the wild type. Five-day-old *scr*-*1* heterozygous plants displayed root meristems shorter than Wt (Fig. 5f) whose roots kept growing over time. On the other hand, *arr1* heterozygous plants displayed root meristems whose size was intermediate between wild-type plants and *arr1* homozygous mutants (Fig. 5f), providing evidence for a dose-dependent control of meristem size by ARR1.

## Discussion

The number of transit-amplifying cells, which undergo a finite number of cell divisions in the proximal meristem (the division zone), can influence the rate of the overall root growth. Therefore, a tight control of the cell number within the meristem tunes the driving force of the root growth.

**Fig. 6:**
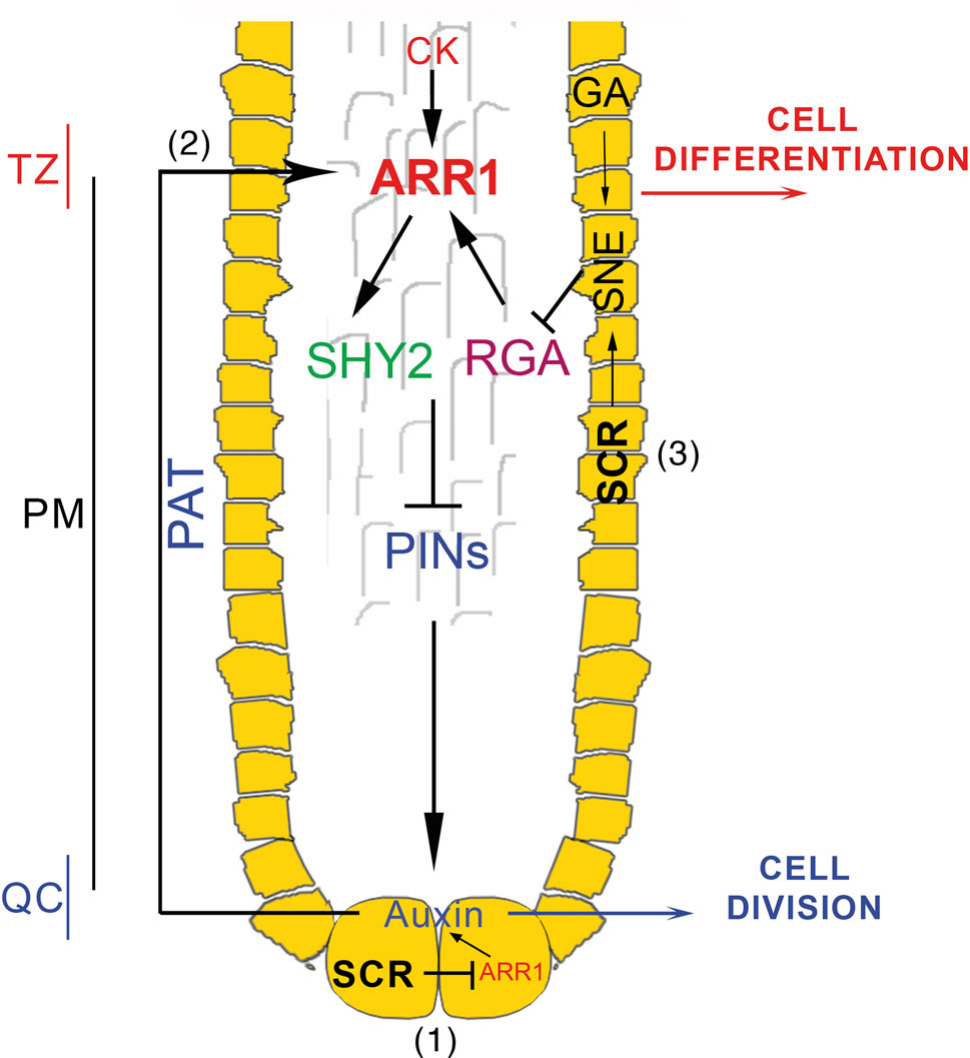
Model for the SCR-based regulatory circuits that control root meristem size. The model shows different tissue-specific activities of SCR on the control of *ARR1* expression. Yellow cells represent SCR expression domain (endodermis and quiescent center, QC). From the QC, SCR represses *ARR1* in two different ways: (1) SCR directly represses *ARR1* in the QC cells, which in turn controls auxin production, thus enabling stem cell division. (2) With the same molecular mechanism SCR, via polar auxin transport (PAT), also exerts a long-distance control on *ARR1* at the transition zone (TZ) enabling cytokinin to sustain cell differentiation via the SHY2/PINs (polar auxin transporters) pathway (Dello Ioio et al. 2008; Moubayidin et al. 2013). (3) From the root endodermis SCR positively transduces gibberellin (GA) signaling, presumably by controlling the F-box SNEEZY (SNE) (Cui et al. 2007), thus in turn controlling the DELLA protein RGA degradation (Ariizumi and Steber 2011; Ariizumi et al. 2011). In this way SCR tightly controls the level of *ARR1*-mediated differentiation, via RGA (Moubayidin et al. 2010 and this work)

Our data unveil a novel, SCR-based, regulatory circuit that controls meristem size from the root endodermis: in this tissue SCR directly activates the gene encoding the F-box protein SNE (Cui et al. 2007) that in the presence of GA mediates the degradation of the DELLA protein RGA (Ariizumi and Steber 2011; Ariizumi et al. 2011) that in turn mediates the regulation of *ARR1* levels at the TZ (Moubayidin et al. 2010) (Fig. 6). This integrates the current view of the control of the Arabidopsis root meristem size by SCR, and allows to propose a general model where the gene SCR is a key regulator of root meristem size acting from two different meristematic tissues on the level of ARR1: in the QC, SCR controls QC and stem cell activities (Sabatini et al. 2003) by directly suppressing the *ARR1* gene (Moubayidin et al. 2013); in addition from the QC, SCR controls stem cell daughters’ differentiation at the TZ by controlling *ARR1* expression at the TZ via auxin (Moubayidin et al. 2013) and from the endodermis via gibberellins (this work, Fig. 6). How, mechanistically, SCR controls *ARR1* expression in different ways from different root tissues will require much further work. An intriguing possibility is that SCR needs different cell/tissue-specific co-factor to sustain its activity as already observed during asymmetric cell division processes necessary to generate the root ground tissues (Cruz-Ramìrez et al. 2012).

### *Author contribution statement*

L.M. conceived the research, planned and performed experiments, analyzed the data and wrote the manuscript; E.S. planned and performed experiments; L.G. performed experiments; I.T. constructed and provided *scr*-*3* N9094 *UAS*::*SCR*::*GR* (*N9094*≫*SCR*:*GR*) seeds; R.H. supervised experiments and provided critical review of the manuscript; P.C. discussed experiments and provided critical review of the manuscript; S.S. conceived the research, analyzed the data and wrote the manuscript.

## Electronic supplementary material

Below is the link to the electronic supplementary material.

Supplementary material 1 (DOCX 342 kb)

## Abbreviations

ARR1: ARABIDOPSIS RESPONSE REGULATOR 1
DEX: Dexamethasone
DPG: Days post-germination
GA: Gibberellin
QC: Quiescent center
RGA: REPRESSOR OF *ga1*-*3*
SCN: Stem cell niche
SCR: SCARECROW
SNE: SNEEZY
TZ: Transition zone
