# Supplementary material for: A SCARECROW-based regulatory circuit controls Arabidopsis thaliana meristem size from the root endodermis
Source: Planta. 2016 Feb 5;243:1159–68. doi: 10.1007/s00425-016-2471-0 (PMC4837209; doi:10.1007/s00425-016-2471-0)
Supplement: Supplementary file 1 — Supplementary material 1 (DOCX 342 kb) [file 425_2016_2471_MOESM1_ESM.docx]

**A Scarecrow-Based Regulatory Circuit Controls *Arabidopsis thaliana* Meristem Size from the Root Endodermis**

**PLANTA**

Laila Moubayidin^1,2^ , Elena Salvi^1^, Leonardo Giustin^1^, Inez Terpstra^3,4^, Renze Heidstra^3,5^, Paolo Costantino^1^, Sabrina Sabatini^1^

*1: Dipartimento di Biologia e Biotecnologie, Laboratory of Functional Genomics and Proteomics of Model Systems, Università La Sapienza - P.le Aldo Moro, 5 - 00185 Rome, Italy*

*2: Present address: Crop Genetics Department, John Innes Centre,* *Norwich Research Park*, *Norwich NR4 7UH, United Kingdom*

*3: Faculty of Science, Department of Biology, section Molecular Genetics, Utrecht University, Padualaan 8, 3584 CH Utrecht, The Netherlands.*

*4:* *Present address: University of Amsterdam, Faculty of Science, SILS POSTBUS 94215, 1090 GE Amsterdam*

*5: Present address: Plant Developmental Biology, Wageningen University and Research Centre, Droevendaalsesteeg 1,6708 PB Wageningen,The Netherlands.*

*Authors for correspondence*

Laila Moubayidin: e-mail: [*laila.moubayidin@jic.ac.uk*](mailto:laila.moubayidin@jic.ac.uk) phone: +441603452135 fax: +441603450027

Sabrina Sabatini: e-mail: [*sabrina.sabatini@uniroma1.it*](mailto:sabrina.sabatini@uniroma1.it) phone: +390649917916 fax:+390649917594

**­­­**

**Supplementary Material**

**
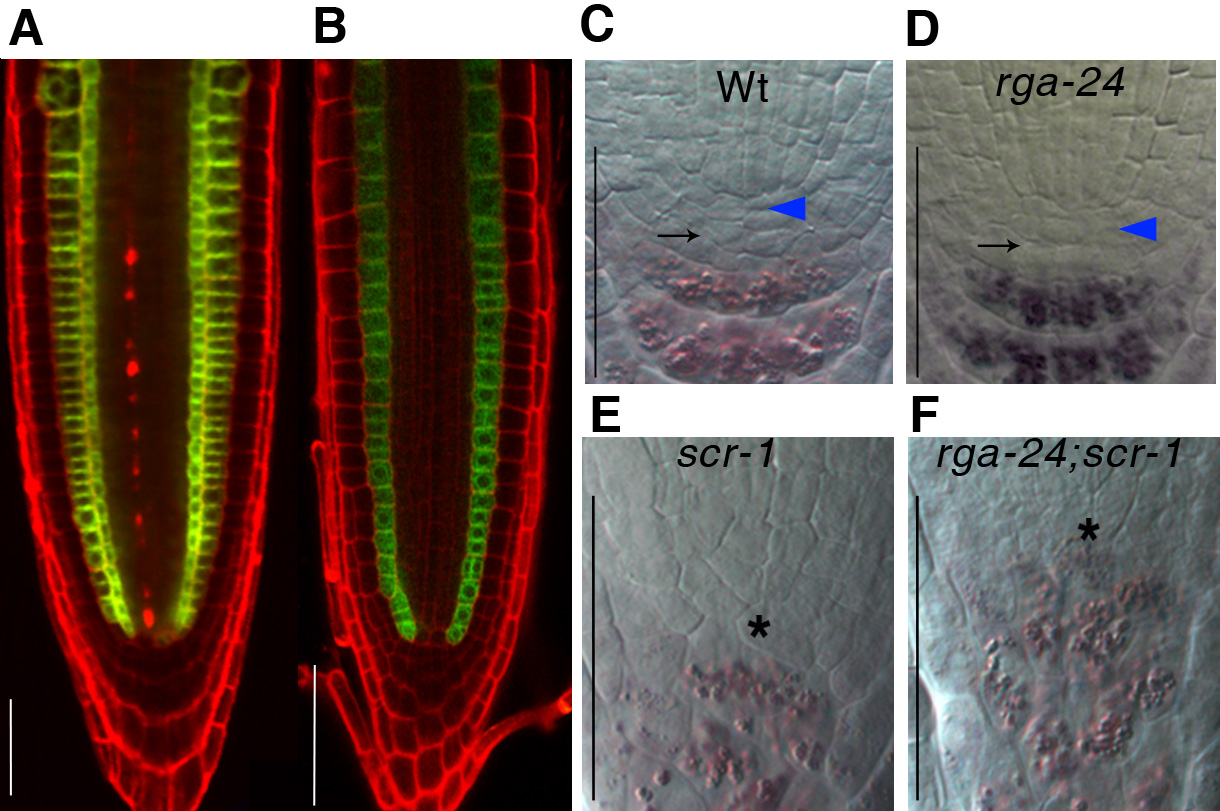
**

**Fig. S1. SCR doesn’t interact genetically with RGA in the control of the stem cell niche activity.** (A,B) Confocal images of the N9094 (*GAL4,UAS∷GFP*) enhancer trap line from the J. Haseloff (http://www.plantsci.cam.ac.uk/Haseloff) collection; (A) in wild-type roots *GFP* is expressed specifically in the cortex and endodermis tissues while (B) in *scr-3* mutant background it marks a monolayer. Scale bars represent 50µm. (C-F) Lugol staining in Wt (C), *rga-24* (D), *scr-1* (E) and *scr-1 rga-24* (F) stem cell niche. The blu arrows point the position of QC, the black arrows indicate columella stem cells in Wt and in *rga-24*, asterisks indicate the position of QC cells in *scr-1* and *scr-1 rga-24* mutants. Scale bars represent 50µm.
